# Differences of corruption types in selected Western and central-eastern health systems during the COVID-19 pandemic: a rapid review

**DOI:** 10.3389/fpubh.2023.1269189

**Published:** 2023-10-09

**Authors:** Alejandro Gonzalez-Aquines, Adolfo C. Cordero-Perez, Iwona Kowalska-Bobko

**Affiliations:** ^1^Institute of Public Health, Faculty of Health Sciences, Jagiellonian University Medical College, Kraków, Poland; ^2^University of Bradford, Bradford, United Kingdom; ^3^School of Health and Related Research, The University of Sheffield, Sheffield, United Kingdom; ^4^Independent Researcher, Zurich, Switzerland

**Keywords:** health corruption, COVID-19, public health crisis, Western Europe, Central-Eastern Europe

## Abstract

**Objectives:**

To identify, describe, and classify the cases of health corruption present in selected Western [the Netherlands and the United Kingdom (UK)] and Central-Eastern European (Poland and Slovakia) countries during the COVID-19 pandemic.

**Methods:**

A rapid review of the literature was conducted, evaluating data from 11 March 2020 to 15 April 2021. Information sources included MEDLINE via WoS, IBSS via ProQuest, Scopus, and gray literature.

**Results:**

Thirteen cases were identified across the four countries. The primary type of health corruption in Western European countries was procurement corruption, while misuse of (high) level positions was the most prevalent in Central-Eastern European countries. Actors from central governments were most involved in cases. The rule of law and anti-corruption watchdogs reported most cases in the United Kingdom and the Netherlands, while the media reported cases in Poland and Slovakia.

**Conclusion:**

The differences in types of corruption in WE and CEE countries emphasize the need to contextualize the approach to tackle corruption. Thus, further research in preventing and tackling corruption is a vital and necessary undertaking despite the inherent of conducting health corruption research.

## Introduction

Corruption is the abuse of entrusted power for private gain and has a significant impact on health systems (1). Corruption costs developing countries over one trillion USD every year. Health care systems (HCSs) themselves lose out on over 500 billion USD annually due to the impact of corruption (2). This impact is not limited to monetary losses, as improvements in the control of corruption result in the more efficient use of healthcare resources. This leads to better outcomes, improving the population’s overall health (3). For instance, Lio and Lee reported that just a 1-point difference in the World Bank’s Control of Corruption Indicator is associated with numerous positive effects. These include a longer life expectancy (0.44 more years), lower rates of infant mortality (2.67 fewer deaths per 1,000 live births), and lower rates of under-five mortality (4.62 fewer deaths per 1,000 children) (4).

Corruption in health systems remains understudied despite its impact on financial and healthcare outcomes. Around half of the literature that combines the terms ‘health’ and ‘corruption’ in PubMed -one of the leading biomedical databases- was produced from 2019 onwards. However, the diversity in terms used to refer to corruption, as well as the lack of mechanisms that protect those reporting corrupt acts, contribute to the difficulty of studying corruption in health systems.

At the same time, corruption tends to increase during periods in which health systems are at their most vulnerable. These include public health crises like the COVID-19 pandemic, in which attention is focused on the response to the crisis. The need for swift action to protect the population compromises the quality of procurement, while modifications in existing processes have unintended consequences on the risk of corrupt practices. This was evident during the COVID-19 pandemic, in which the nature of procurement changed dramatically. For example, reduced scrutiny in the face of necessity led to actors from healthcare systems engaging in corrupt practices (5).

Social and cultural context are additional elements to consider in the study of corruption in health systems. Societies’ perception of corruption varies from country to country, imposing challenges in the reporting of corrupt practices. An example of this is the perception of giving gifts to obtain something from public service, with Central and Eastern European (CEE) countries having a higher acceptance of this than Western European (WE) countries (6). The differences in corruption between WE and CEE countries are also evident in corruption performance indicators. Take, for instance, the Control of Corruption Index (measured from 0 to 100 - the higher the number, the better the control of corruption) by the World Development Indicators. This showed that the United Kingdom and the Netherlands scored 94 and 96 respectively, while Poland and Slovakia scored 73 and 66. Similarly, we can look at the rule of law, which can be understood as “the principle that political power must be exercised in accordance with law rather than in an arbitrary or self-interested manner, and that disputes among private individuals and between them and the Sovereign must be subjected to independent adjudication.” (7) The Rule of Law Index (measured from 0 to 1 - the closer to zero, the lower the adherence to the rule of law) captures compiles data from nine factors to provide a quantitative measure of this principle (7). According to this measure, it is evidenced that the rule of law is higher in the United Kingdom and the Netherlands (0.82 and 0.88 respectively) when compared to Poland and Slovakia (0.73 and 0.51 respectively) (8).

While there are available reports from CEE and WE countries of corrupt practices during the COVID-19 pandemic, most of these are presented as single cases from local facilities (9), narrative reviews (10), or evaluations of a country’s procurement by anti-corruption organizations (5, 11). These reports provide an important contribution to the field. However, there is a need for a systematic evaluation that considers the context of individual countries. This will enable the identification of corrupt practices that might not be perceived as such in other societies.

It is important to contextualize the evaluation of corrupt practices during crises. This arises from the need to create policies that will prevent and promptly identify the most prevalent types of corruption in each individual country. Moreover, the study comes at a time of poly-crises; namely the Russian invasion of Ukraine, the cost-of-living crisis, and the worsening impacts of climate change (12). These factors all highlight the need to ensure that resources are used efficiently to protect the population’s health.

This study acknowledged the differences between the perception and the state of corruption in WE and CEE countries, together with the increased vulnerability of health systems to corruption during public health crises. Consequently, the aim was to identify, describe, and classify the cases of health corruption present in selected Western and Central-Eastern European countries during the COVID-19 pandemic.

### The vulnerability of health systems and its actors to corruption

**Figure 1:**
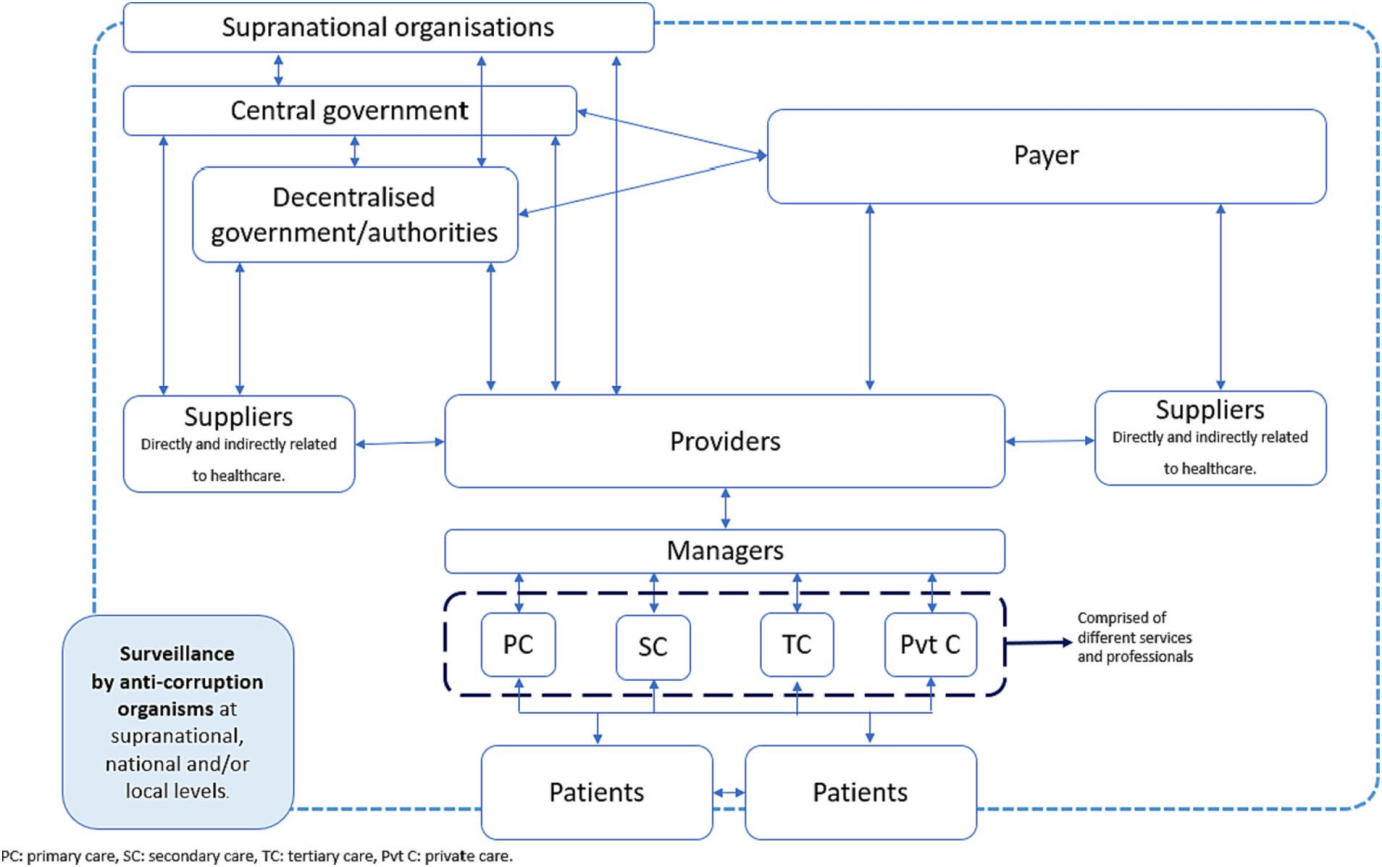
Interactions between actors from the health system (8).

Health systems are defined as the actors (organizations, institutions, people) whose primary goal is to improve health (13). In order to achieve this goal, actors engage in interactions both inside and outside the health system (Figure 1). For instance, the COVID-19 pandemic required both the redesign of existing hospitals and the creation of new ones. This involved not only the healthcare sector, but also the construction, financial, and environmental sectors as well.

While health system actors aim to improve health, these interactions between sectors occur under an asymmetric distribution of information, also known as the principal-agent (PA) problem. In context of healthcare, the PA problem refers to the provider (agent) of a service maximizing profits at the expense of the actor in the system (principal) (14). An example of how the PA problem was present during the COVID-19 pandemic is the allocation of financial resources to car manufacturers (*agent*), who falsely claimed to be able to provide ventilators to health systems (*principal*) (15).

In addition to the PA problem, other factors contribute to the occurrence of corrupt practices in health systems. Vian developed a theoretical framework that illustrates how the interactions between health system actors lead to the abuse of entrusted power for private gain (16). The framework considers three main factors that influence actors to engage in corrupt practices:

1. Rationalization.
2. Opportunity to abuse.
3. Pressure to abuse.

Rationalization refers to behavior influenced by social norms and ethical beliefs. The opportunity to abuse is influenced by a country’s health system structure, and includes the level of monopoly, discretion, accountability, citizen voice, transparency and enforcement. Lastly, the pressure to abuse stems from pressure from clients, as well as that from wages or incentives. Based on Vian’s theoretical framework, we hypothesized that the structural and social differences between WE and CEE countries would yield to different types of corruption during the COVID-19 pandemic.

## Methods

The highly dynamic pace of the pandemic required a fast approach to studying how health corruption unfolded through the different waves. Therefore, a rapid review of the literature was considered the most appropriate approach. This study followed the practical guide for rapid reviews by the World Health Organization (17). An internal protocol (available upon request) for the rapid review was developed prior to the conduction of the review. However, this was not registered on PROSPERO, as it is not yet possible to register rapid reviews on this website.

Although the methodology adhered to these guidelines, it is worth emphasizing that corruption is a complex topic to research due to imbalances in power. This can lead to actors being reluctant to report corruption cases, or cases not being judicially solved due to a weak rule of law (18). Therefore, while the conducted methodology did provide an indication of how corruption unfolded in the selected countries, the authors acknowledge that it did not guarantee that all corruption cases were identified.

This review included four countries, two from WE and two from CEE. These countries were selected based on their geographical location (Western vs. Central-Eastern Europe), the contrast in corruption indicators (control of corruption index and rule of law index), the availability of information in English or Polish, and finally the authors’ ability to contact experienced health experts from these countries to help identify additional cases.

The inclusion criteria for identified studies were determined following the CoCoPop (Condition, Context, Population) approach (19). Although CoCoPop was created for observational studies evaluating the prevalence or incidence of a specific disease, the authors considered it appropriate for the research’s aim. This was because it aligned with the aspects under investigation. The elements of this mnemonic are stated as follows:

- Co: Health corruption.
- Co: Covid-19 pandemic.
- Pop: Selected WE and CEE countries.

In accordance with CoCoPop, the included studies must have addressed health corruption (condition) in any form. The European Commission typology (Table 1) was used to classify the cases of corruption, as the European Commission serves as a supranational ruling authority for all included countries. This ensured a comprehensive and standardized approach. For the context element, studies or reports must have been performed in the context of the COVID-19 pandemic. They must also have reported health corruption in an activity related to either preparedness for the pandemic or the response to it. For the population element, eligible studies referred to the selected WE (the Netherlands and the United Kingdom) and CEE (Poland and Slovakia) countries. The timeframe was limited to the period between March 11th 2020, when the WHO declared COVID-19 a pandemic (21), and April 15th 2021, when the last literature search was conducted. Only studies published in English and Polish were considered.

**Table 1:** Types of health corruption included in the review.

Corrupt practice	Subtypes
Bribery in medical service delivery	Access to healthcare Preferential treatment Better quality of healthcare False sick leave statements
Procurement corruption	Pre-bidding: corruptive needs assessment Pre-bidding: circumvention of tender procedures Pre-bidding: tailored tendering Bidding: bribery and kickbacks during the bid evaluation Bidding: favoritism Bidding: collusion and/or market division in bidding Post-bidding: false invoicing Post-bidding: changing contract agreements
Improper marketing relations	Direct prescription influencing (quid-pro-quo deals) Indirect prescription influencing (creation of loyalty) Undue positive list promotion Authorization of medicines and certification of medical devices
Improper marketing regulations	
Misuse of (high-level) positions	Revolving door corruption Regulatory state capture Trading in influence Conflict of interest Favoritism and nepotism
Undue reimbursement claims	‘Upcoding’ (reimbursement of maximum tariffs) Reimbursement of unnecessary treatments Reimbursement non-delivered treatments
Fraud and embezzlement of medicines and medical devices	Sale of public or prepaid medicines for private gain Sale of counterfeit medicines Use of publicly owned or financed devices or facilities for private gain

Source: Study on Health Corruption by the European Commission (20).

The study types that were considered for inclusion included abstracts, observational studies (e.g., case studies and case reports), and reports available in the gray literature (policy briefs, statements from governmental and non-governmental organizations, news, and media reports). Health and politics-related databases were also consulted. These included Scopus, MEDLINE *via* WoS, and IBSS (International Bibliography of the Social Sciences) *via* ProQuest. Additional sources of information were also used, such as Transparency International and its relevant national chapters, the European Commission for reports from the Netherlands, Poland and Slovakia, and Anti-Corruption United Kingdom for information from the United Kingdom. As health corruption usually becomes known through media scandals, a Google search was also performed and limited to the first 50 results. Furthermore, we used the Web of Science to examine the references and track the citations of all included studies. Finally, we contacted health system experts from the four countries to ask for cases not identified through the literature search.

The following groups of terms and synonyms were considered for the search strategy: Health corruption, bribery, extortion, fraud, nepotism, racketeering; COVID-19, SARS-CoV-2, coronavirus; and The Netherlands, Poland, Slovakia, the United Kingdom. The search strategies for MEDLINE, IBSS, and Scopus, are presented in the Supplementary Tables S1–S3. An advanced Google search was performed using the same terms. Google Chrome’s Incognito Mode was used during the Google search to avoid saved cookies and browsing history.

Two authors collaborated in the selection of the studies. AGA conducted the search strategy and selected studies for potential inclusion. Titles, abstracts, and full-text articles were then screened and evaluated by two authors (AGA and ACP). Decision disagreements were solved on consensus or by a third author (IKW). A PRISMA flowchart of the studies was constructed to illustrate the study selection flow. Due to the nature of reporting corruption cases, the authors anticipated most cases would be identified in gray literature, for which standard risk of bias assessments (e.g., ROBINS-I, Newcastle-Ottawa) would not be appropriate. Therefore, the Authority, Accuracy, Coverage, Objectivity, Date, and Significance (AACODS) checklist was conducted by two authors (AGA and ACP). This served to conduct a critical evaluation and determine the risk of bias from the identified studies (22). A third author (IKW) was consulted when discrepancies occurred.

The extracted information included: 1) a description of the case, 2) the country where it occurred, 3) the classification of health corruption using the European Commission framework (Table 1), 4) the actor from the HCS who was involved in the case based on Figure 1, 5) the date when the case occurred, 6) the date the case was acknowledged or identified, and 7) the person/organization who acknowledged or made the case public. One author (AGA) extracted the information, which was later reviewed by ACP. Discrepancies in the data extraction were achieved through consensus. Data synthesis was performed using the Synthesis Without Meta-analysis (SWIM) guidelines (23). The narrative synthesis was performed around the following themes: the types of health corruption identified (10), the actor(s) involved in the identified cases, and the actor(s) that made the case public.

## Results

**Figure 2:**
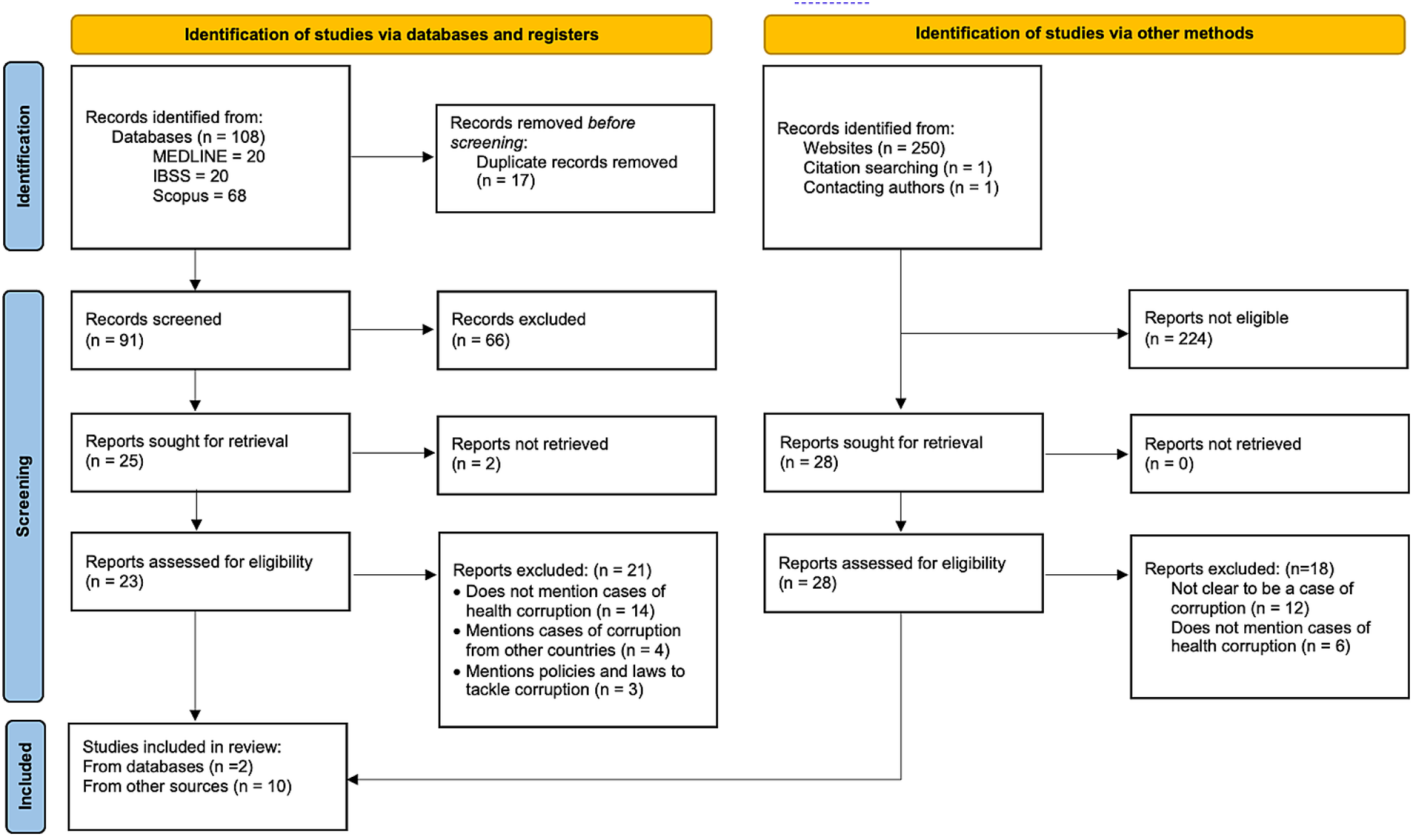
PRISMA flowchart of the included studies. Adapted from Page et al. (24).

Twelve studies were included in the final analysis. A critical appraisal of the included studies and the reference list of the excluded studies is presented in Supplementary Tables S4 and S5 of the supplementary material. Most were found through gray literature and the rest from opinion articles (Figure 2). From these, 13 cases were identified. The majority were from United Kingdom (6 cases - 46.2%), followed by Poland (4 cases - 30.8%), Netherlands (2 cases - 15.4%), and Slovakia (1 case - 7.7%). A summary of the description of the identified cases is shown in Table 2 and the results of the AACODS checklist for critical appraisal is presented in Table 3.

**Table 2:** Summary of findings from the included studies.

Author, year Country	Description of the case	Actor(s) of the HCS involved	Classification of health corruption	Date of occurrence and announcement	Responsible for the announcement
Kość (25) Poland	The public health minister spent 5 million złoty (€1.1 million) on 120,000 FFP-2 type face masks and 20,000 surgical masks that were later found not to meet safety standards. The contract originated from a ski instructor who knew the actor’s brother, a health care businessman.	Central government, Health Ministry	Fraud and embezzlement of medicines and medical devices. Misuse of (high-level) positions.	Occurrence: Not clear Announcement: Not clear	Newspaper, Wyborcza
Koper (26) Poland	A former deputy health ministry agreed to pay €35 million to an arms dealer for ventilators without experience in health devices. Normally, the authorities hold public tenders for such large deals. In March, Poland’s parliament passed a law saying that tenders are unnecessary in COVID times.	Central government, Deputy Health Ministry	Fraud and embezzlement of medicines and medical devices. Misuse of (high-level) positions.	Occurrence: April 14th, 2020 Announcement: December 17th, 2020	Newspaper, Reuter
ENCA (27) Poland	Politicians, including members of the ruling Law and Justice (PiS), a Member of the European Parliament, and the former Prime Minister, received their COVID-19 vaccine ahead of their turn.	Supranational organization, Member of European Parliament. Central government, members of the ruling party.	Misuse of (high-level) positions.	Occurrence: December 30th, 2020 Announcement: January 4th, 2021	Newspaper, ENCA
Central Anti-corruption Bureau (28) Poland	Between March and June 2020, the Director of the Military Institute of Hygiene and Epidemiology in Warsaw abused his powers by appropriating property belonging to the Institute. This property included protective equipment, disinfectant fluids, and tests for COVID-19. The investigation indicates that those items were then transferred to unauthorized persons.	Central government institution, Director of the Military Institute of Hygiene and Epidemiology in Warsaw.	Misuse of (high-level) positions	Occurrence: Not clear Announcement: Not clear	Central Anti-corruption Bureau
Kern (29) Slovakia	The Head of the Material Reserves office purchased 200,000-speed tests of lower quality for the novel coronavirus for €6 million, 15 times more than what China paid. Also, he received a €200,000 transfer to his account, suspected to be from bribery.	Central government, Head of the Material Reserves	Procurement corruption Misuse of (high-level) positions	Occurrence: April 21st, 2020 Announcement: April 21st, 2020	Police from Slovakia
Interpol (30) Netherlands	Interpol detected a scheme to defraud German authorities in transactions to purchase face masks. A Dutch supplier requested €1.5 million in advance, claiming that the funds were not transferred, and an additional €880,000 were demanded to secure the shipment.	Supplier, a Dutch company.	Fraud and embezzlement of medicines and medical devices	Occurrence: March 15th, 2020 Announcement: April 15th, 2020	Interpol
Homolova and Lyndel (31) Netherlands	Information about the contracts awarded for purchasing goods related to the COVID-19 pandemic has not been made public. This includes the names of the companies awarded the contracts and the amount received.	Central and/or decentralized government.	Procurement corruption	Occurrence: Not clear Announcement: October 21st, 2020	Organized Crime and Corruption Reporting Project
Goodrich (32) United Kingdom	A total of 24 PPE contracts, accounting for £1.6 billion, were awarded to those with known political connections to the Conservative Party, and three contracts worth £536 million went to politically connected companies for coronavirus testing services.	Central government members of the ruling party.	Misuse of (high-level) positions. Procurement corruption.	Occurrence: February 2020 to December 2020 Announcement: April 22nd, 2021	Transparency International
National Audit Office (33) United Kingdom	“PPE suppliers with political connections were directed to a “high-priority” channel for United Kingdom government contracts where bids were ten times more likely to be successful. Almost 500 suppliers with links to politicians or senior officials were referred to the channel, where their pitches for contracts were automatically treated as credible by government officials charged with procuring PPE.	Central government, Department of Health and Social Care, Department of Education, and Cabinet Office	Misuse of (high-level) positions. Procurement corruption.	Occurrence: From March 2020 to July 2020 Announcement: November 26th, 2020	National Audit Office
BBC (34) United Kingdom	A court ruled that the Secretary of State for Health and Social Care acted unlawfully by not revealing the information about the contracts it had signed during the COVID-19 pandemic.	Central government, Secretary of State for Health and Social Care	Procurement corruption.	Occurrence: From March 2020 to December 2020 Announcement: February 19th, 2021	High court
Kohler and Wright (9), United Kingdom	A senior NHS official in London working at the capital’s Covid-19 Nightingale hospital launched a business in April 2020 to trade PPE. The official was suspected to be part of procurement for PPE in the NHS.	Decentralized authority, NHS official	Misuse of (high-level) positions. Procurement corruption.	Occurrence: April 15th, 2020 Announcement: May 1st, 2020	News, The Guardian
Kohler and Wright (9) United Kingdom	Public Health England paid for antibody kits for COVID-19, but they later proved inaccurate.	Central government, Public Health England	Fraud and embezzlement of medicines and medical devices.	Occurrence: April 1st, 2020 Announcement: April 11th, 2020	Greg Clark, Chair of the Commons science and technology committee
Armstrong (35) United Kingdom	The United Kingdom government awarded a contract of £75 m for one million antibody tests to a business consortium. A public tender was not carried out, and the accuracy of the tests was not evaluated.	Decentralized authorities, United Kingdom Rapid Test Consortium	Fraud and embezzlement of medicines and medical devices. Procurement corruption.	Occurrence: October 6th, 2020 Announcement: November 12th, 2020	Good Law Project

PPE, Personal protective equipment.

**Table 3:** AACODS critical appraisal based.

	Authority	Accuracy	Coverage	Objectivity	Date	Significance
Kość (25)	?	Y	Y	Y	Y	Y
Koper (26)	Y	Y	Y	Y	Y	Y
ENCA (27)	N	?	Y	Y	Y	Y
Central Anti-corruption Bureau (28)	Y	Y	Y	Y	Y	Y
Interpol (30)	Y	Y	Y	Y	Y	Y
Homolova and Lyndel (31)	Y	N	?	?	?	Y
Kern (29)	Y	Y	Y	Y	Y	Y
Goodrich (32)	Y	Y	Y	Y	Y	Y
National Audit Office (33)	Y	Y	Y	Y	Y	Y
BBC (34)	Y	Y	Y	Y	Y	Y
Kohler and Wright (9)	Y	Y	Y	Y	Y	Y
Kohler and Wright (9)	Y	Y	Y	Y	Y	Y
Armstrong (35)	Y	Y	Y	Y	Y	Y

### Types of health corruption identified

Three main types of health corruption were identified within the 13 cases: misuse of (high-level) positions (8 out of 13 cases); procurement corruption (7 out of 13 cases); and fraud and embezzlement of medicines and medical devices (5 out of 13 cases). In over half of the cases (53.8%), more than one type of health corruption was present. In WE countries, procurement corruption was present in 6 out of 8 cases. Fraud and the embezzlement of medicines/medical devices were reported in 3 out of 8 cases, while misuse of (high-level) positions also occurred in 3 out of 8 cases.

In the United Kingdom, for example, the National Audit Office reported that the UK government produced a “high-priority” list of suppliers with political connections (36). At the same time, cases of fraud were present as Public Health England paid for antibody kits that were later confirmed to be inaccurate (9). In the Netherlands, a case of procurement corruption was reported by Homolova and Lyndell (31), while Interpol detected an instance of fraud involving €1.5 million (30).

Contrastingly, CEE countries reported a higher number of cases in which the misuse of (high-level) positions was present (5 out of 5 cases). Fraud and the embezzlement of medicines /medical devices were present in 2 out of 5 cases, while procurement corruption was found in 1 out of 5 cases. Examples of the misuse of (high-level) positions occurred in Poland at the Military Institute of Hygiene and Epidemiology in Warsaw, as reported by the Central Anti-Corruption Bureau (28). In Slovakia, the Head of the Material Reserves acquired COVID-19 tests at 15 times the original price (29).

### Actors of the HCS involved

Most of the actors involved in health corruption cases identified during the COVID-19 pandemic were authorities or organizations at central government level (9 out of 13 cases). This was followed by decentralized authorities (5 out of 13 cases), while only one supplier and one actor at the supranational level were involved among all 13 cases.

The actors at central government level were the most commonly involved in both WE (5 out of 8 cases) and CEE countries (5 out of 5 cases). Decentralized authorities were involved in one case in WE (9). An authority from a supranational organization was involved in one case in Poland (27), while a supplier was responsible for a case of health corruption in the Netherlands (37).

### Authority or organization that made the case public

From the 13 cases, four were reported by the news, three by the rule of law authorities, three by non-governmental anti-corruption organizations, two by the police, and one by a government anti-corruption watchdog. The organization that made the cases public differed between WE and CEE countries. For instance, in the United Kingdom and the Netherlands, three cases were reported by an anti-corruption NGO (Good Law Project, Transparency International, and the Organized Crime and Corruption Reporting Project), three by the rule of law (National Audit Office, Chair of the Commons, and High Court), one by the media, and one by the Interpol. In CEE, the authorities reporting cases of corruption during the COVID-19 pandemic were mainly the media (3 out of 5 cases), one by the police, and only one by an anti-corruption watchdog (28).

## Discussion

Our research presented the results of a rapid review of the literature used to identify, describe, and categorize health corruption cases in WE and CEE countries during the COVID-19 pandemic. The findings showed that corruption was present in each of the studied countries. Evaluations of corruption during the pandemic are still emerging among European countries, with the number of reports expected to increase as data on procurement and resource allocation is analyzed by researchers, media, and watchdogs. Recent literature in the field has explored the impacts of corruption on public trust and mortality due to COVID-19 (37, 38).

As of the writing of this article, Europe faces fresh shocks in the form of the war in Ukraine, the refugee flow, and the need to create decent and equitable conditions of stay for the individuals concerned. As demonstrated during the COVID-19 pandemic, each crisis increases the potential for corrupt activities. Studies in this area are extremely important, particularly as cases of corruption related to these crises continue to be brought to light (39, 40).

Our review found the misuse of (high-level) positions to be the most prevalent type of health corruption in CEE countries during the COVID-19 pandemic. Procurement corruption was the main type encountered in WE countries. This was also the case prior to the pandemic (41). The involvement of actors from high-level positions in CEE countries is well documented in the literature. It is explained by the small, tight-knit friendship networks formed in the political sphere during the communist and post-communist eras, which facilitated opportunities to engage in these practices without being penalized (42). Meanwhile, better enforcement of the rule of law might explain the higher prevalence of procurement corruption found in WE countries (43).

The OECD has identified public procurement as one of the most vulnerable governmental activities. This is due to the high volume of transactions, financial interests, the complexity of the process, close interaction between public and private sector officials, and the multiple stakeholders involved (44). In the context of the COVID-19 pandemic, procurement was one of the first actions taken by the government to obtain the medical equipment required to address the pandemic (i.e., personal protective equipment (PPE), ventilators, etc.). Not surprisingly, most health corruption cases occurred when the government acquired these goods. In the United Kingdom alone, Transparency International identified 65 “questionable contracts” geared toward acquiring PPE, for which the government paid £2.9 billion (11). In addition to the inherent complexity of public procurement, governments approved laws that reduced the transparency of purchases during the pandemic. For instance, in the United Kingdom, the government introduced a “high-priority” channel for government contracts (9). In Poland, policies were implemented to give impunity for decisions taken to protect the population during the COVID-19 pandemic, even if these actions were not legal (45).

It is easy to understand the need to streamline the process for procuring medical goods during the COVID-19 pandemic, as it helped to ensure a rapid response to the crisis. However, corruption’s long-term negative impact on the health sector might eventually outweigh the benefits, resulting in a lack of trust from the public sector. The consequences of distrust are directly associated with the public’s behavior in response to a crisis. This was shown by Han et al., who reported that higher trust in government was reflected in the public’s compliance with COVID-19 protection measures (handwashing, avoiding crowded spaces, self-quarantine, etc.). This also shows the fundamental importance of strong levels of trust before the onset of a crisis. As evidenced during the pandemic, the populations of countries with higher trust at baseline were far more likely to follow the recommended safety measures (46).

In all four countries, actors from the central government were the main ones involved in the identified cases. However, the mechanisms to report these cases differed among WE and CEE countries. Anti-corruption organizations reported most cases in WE countries, while media scandals were most the most frequent method of unearthing corruption in CEE countries. The centralized use of resources might explain the central government’s involvement in most cases. This is shown by the recorded subnational government spending in each country: less than 10% in the Netherlands, Slovakia and the United Kingdom, and less than 20% in Poland (47).

The close networks of friends among the political authorities might hinder the reporting of health corruption in CEE, thus making it necessary to establish effective whistleblowing mechanisms (38). Efforts to improve whistleblowing and protect those reporting corruption have been made at the supranational level, as evidenced by the European Parliament’s Directive on protecting persons who report breaches of Union Law. This directive was expected to be adopted by all member states before the end of 2021. However, at the time of writing (August 2022), only 11 out of the 27 member states have adopted this law. The Netherlands, Poland and Slovakia are among those delaying its implementation (48).

### Implications for policy and research

The implications for policy include the need to create appropriate anti-corruption mechanisms, as well as to implement anticipatory mechanisms for the rapid identification of cases of corruption. These anti-corruption mechanisms should prioritize safe whistleblowing to ensure that health system actors are protected and empowered to report corrupt practices. It is also important to enhance transparency and accountability for public procurement. A solution that has been successfully implemented in other European countries is open contracting for health (OC4H). OC4H is grounded in the wisdom that prevention is better than cure, and that the beneficiaries of the procurement (citizens, populations, patients) are in a better position to monitor the process (49). Lastly, anticipatory governance also plays a vital role in the prevention of corruption. Anticipatory governance aims to foresee and prevent the (unintended) negative consequences of policies using a whole-of-system and whole-of-government approach (50).

One implication for future research is found in the role our study played in identifying and categorizing cases of corruption, while also highlighting the differences between Western and Central-Eastern European countries. Our study brought into focus the contrast between both regions, demonstrating the need to individually tailor the ways we study and tackle corruption to best suit each country. Moreover, as cases of corruption continue to be reported, comprehensive reviews involving greater numbers of countries are needed. Reviews could be complemented by interviews with stakeholders, helping to identify further challenges and solutions regarding the uncovering and reporting of corruption.

### Limitations

This study was constrained by notable limitations and the findings should be interpreted with caution due to the nature of the topic. We followed a systematic approach using standardized methods to explore a complex subject. However, the political implications of corruption and its sensitivity pose significant limitations to this study and following a systematic approach is not sufficient to capture all the corruption cases during the studied period. Thus, the study’s results should be taken as additional research that contributes with evidence to the field of healthcare corruption rather than a solid conclusion on the topic. As mentioned previously, the nature of corruption makes it challenging to identify and report in the first place. The number of cases is limited due to their political implications and the fear of consequences and retaliation to report the cases. This limitation is widely recognized in the field of corruption in the healthcare sector (51). Publication bias places an important role in this matter. Although in health research publication bias refers to the selective publication of findings (52), in the study of health corruption this is related to the overall lack of reporting due to the safety implications mentioned before. Moreover, the review was conducted during the early stages of the pandemic, which might provide a limited picture of the overall number of cases. This is because, as stated earlier, new cases are expected to be reported as governments’ decisions are scrutinized more closely.

Additionally, all information was limited to the English and Polish languages. Although we tried to overcome this limitation by contacting health system experts with experience in corruption research from each studied country to help detect additional cases, there is no guarantee that the experts were aware of all the cases. Therefore, the findings from non-English and non-Polish speaking languages should be interpreted cautiously as we might have missed other corruption cases. Finally, we did not explore the juridical verdict of the identified cases, as these processes are usually lengthy, and their outcomes depend mainly on the country’s rule of law. Instead, we limited our analysis to identifying, describing, and categorizing corruption cases in the studied countries.

## Conclusion

Cases of corruption in the health care sector were present in all four studied countries. However, the types of corruption differed in each country, with a higher prevalence of procurement corruption in WE countries and misuse of high-level positions among CEE. While a rapid response is necessary to deal with a shock like the COVID-19 pandemic, countries’ efforts should focus on increasing the health systems’ resilience by ensuring adequate resources and tackling corruption. As other crises emerge across Europe, corruption threatens countries’ success in implementing effective responses. Thus, further research in preventing and tackling corruption is a vital and necessary undertaking despite the inherent limitations of conducting health corruption research.

## Author contributions

AG-A: Conceptualization, Formal Analysis, Investigation, Methodology, Validation, Writing – original draft. AC-P: Data curation, Formal Analysis, Writing – review & editing. IK-B: Investigation, Supervision, Validation, Writing – review & editing.
