# Supplementary material for: Differences of corruption types in selected Western and central-eastern health systems during the COVID-19 pandemic: a rapid review
Source: Front Public Health. 2023 Oct 9;11:1269189. doi: 10.3389/fpubh.2023.1269189 (PMC10590903; doi:10.3389/fpubh.2023.1269189)
Supplement: Supplementary file 1 [file Table_1.docx]

**Supplementary documents**

Table S1. Results of search strategy from Medline via OvidSP

Table S2. Results of search strategy from IBSS via ProQuest

Table S3. Results of search strategy from Scopus.

Table S4. Critical appraisal to the included articles.

Table S5. Excluded references from databases

**Table S1. Results of search strategy from Medline via OvidSP**

| 1 | health corruption.mp. | 8 |
| --- | --- | --- |
| 2 | bribery.mp. | 127 |
| 3 | extortion.mp. | 141 |
| 4 | exp Fraud/ | 7358 |
| 5 | nepotism.mp. | 136 |
| 6 | racketeering.mp. | 29 |
| 7 | 1 OR 2 OR 3 OR 4 OR 5 OR 6 | 7748 |
| 8 | exp COVID-19/ | 71 160 |
| 9 | exp SARS-CoV-2/ | 55 232 |
| 10 | exp Coronavirus/ | 67 590 |
| 11 | 8 OR 9 OR 10 | 84 051 |
| 12 | 7 AND 11 | 20 |

Step 7 combines the different terms for health corruption using ‘OR’.

Step 11 combines the different terms for COVID-19 using ‘OR’

Step 12 combines steps 7 and 11 using ‘AND’.

**Table S2. Results of search strategy from IBSS via ProQuest**

| 1 | noft(health corruption OR bribery OR extortion OR fraud OR nepotism OR racketeering) | 10 346 |
| --- | --- | --- |
| 2 | noft(covid-19 OR coronavirus OR sars-cov-2) | 5 917 |
| 3 | 1 AND 3 | 20 |

Step 3 combines the different terms for health corruption and COVID-19 using ‘AND’

**Table S3. Results of search strategy from Scopus.**

| 1 | TITLE-ABS-KEY (“health corruption” OR bribery OR extortion OR fraud OR nepotism OR racketeering) | 34 823 |
| --- | --- | --- |
| 2 | TITLE-ABS-KEY(covid-19 OR coronavirus OR sars-cov-2) | 163 555 |
| 3 | 1 AND 3 | 68 |

Step 3 combines the different terms for health corruption and COVID-19 using ‘AND’

**Table S4. Critical appraisal to the included articles.**

|  | **Authority** | **Accuracy** | **Coverage** | **Objectivity** | **Date** | **Significance** | **Rationale** |
| --- | --- | --- | --- | --- | --- | --- | --- |
| **Czuchnowski W. 2020** | ? | Y | Y | Y | Y | Y | The report was published by a media that has been considered bias in the material produced.^[[1]](#footnote-1)^ |
| **Kopper A., 2020** | Y | Y | Y | Y | Y | Y | NA |
| **ENCA, 2021** | N | ? | Y | Y | Y | Y | It is not clear who the author of the report is. Also, the report is mainly based on a social media complaints that politicians receiving the vaccine out of turn |
| **Central Anti-corruption Bureau, 2020** | Y | Y | Y | Y | Y | Y | NA |
| **Interpol** | Y | Y | Y | Y | Y | Y | NA |
| **Homolova A. and Lyndell D, 2020** | Y | N | ? | ? | ? | Y | Although the authors provide a link to access the data used in their report, these are not available due to an website error. |
| **Kern M., 2020** | Y | Y | Y | Y | Y | Y | NA |
| **Goodrich S., 2021** | Y | Y | Y | Y | Y | Y | NA |
| **National Audit Office, 2020** | Y | Y | Y | Y | Y | Y | NA |
| **BBC, 2021** | Y | Y | Y | Y | Y | Y | NA |
| **Kohler, J.C., Wright, T., 2020** | Y | Y | Y | Y | Y | Y | NA |
| **Kohler, J.C., Wright, T., 2020** | Y | Y | Y | Y | Y | Y | NA |
| **Armstrong S., 2020** | Y | Y | Y | Y | Y | Y | NA |

**Table S5. Excluded references from databases**

|  | Reference | Reason for exclusion |
| --- | --- | --- |
| 1 | Tax evasion, corruption and COVID-19 health risk exposure: a cross country analysis.   A. E. Yamen  Journal of Financial Crime 2021 | Does not mention cases of health corruption |
| 2 | Measures to strengthen primary health-care systems in low- and middle-income countries  E. V. Langlois, A. McKenzie, H. Schneider and J. W. Mecaskey  World Health Organization. Bulletin of the World Health Organization 2020 Vol. 98 Issue 11 Pages 781-791 | Does not mention cases of health corruption |
| 3 | COVID-19 and the need for stringent rules on data sharing  . K. Krishan and T. Kanchan  Acta Biomedica 2021 Vol. 92 Issue 1 | Does not mention cases of health corruption |
| 4 | Uses and abuses of real-world data in generating evidence during a pandemic  K. Khunti, F. Zaccardi, N. Islam and T. Yates  Journal of the Royal Society of Medicine 2021 Vol. 114 Issue 3 Pages 109-110 | Does not mention cases of health corruption |
| 5 | On the authenticity of COVID-19 case figures  . A. P. Kennedy and S. C. Phillip Yam  PLoS ONE 2020 Vol. 15 Issue 12 December | Does not mention cases of health corruption |
| 6 | Combatting Corruption and Collusion in UK Public Procurement: Proposal for Post-Brexit Reform  A. Jones  Modern Law Review 2021 | Does not mention cases of health corruption |
| 7 | Interpretable Sentiment Analysis based on Deep Learning: An overview.   S. Jawale and S. D. Sawarkar  2020 Pages 65-70 | Does not mention cases of health corruption |
| 8 | COVID-19: Fear, quackery, false representations and the law  I. Freckelton Qc  International journal of law and psychiatry 2020 Vol. 72 Pages 101611 | Does not mention cases of health corruption |
| 9 | Global health and human rights in the time of COVID-19: Response, restrictions, and legitimacy  L. Forman and J. C. Kohler  Journal of Human Rights 2020 Vol. 19 Issue 5 Pages 547-556 | Does not mention cases of health corruption |
| 10 | The Corruption of Science.   B. A. Chabner  Oncologist 2020 Vol. 25 Issue 11 Pages 907-908 | Does not mention cases of health corruption |
| 11 | Cybercrime and shifts in opportunities during COVID-19: a preliminary analysis in the UK  D. Buil-Gil, F. Miró-Llinares, A. Moneva, S. Kemp and N. Díaz-Castaño  European Societies 2021 Vol. 23 Issue S1 Pages S47-S59 | Does not mention cases of health corruption |
| 12 | Frauds in scientific research and how to possibly overcome them  E. Boetto, D. Golinelli, G. Carullo and M. P. Fantini  Journal of Medical Ethics 2020 | Does not mention cases of health corruption |
| 13 | Covid-19: A window of opportunity for positive healthcare reforms  S. Auener, D. Kroon, E. Wackers, S. van Dulmen and P. Jeurissen  International Journal of Health Policy and Management 2020 Vol. 9 Issue 10 Pages 419-422 | Does not mention cases of health corruption |
| 14 | The rise of telemedicine: how to mitigate potential fraud.   P. Pointer  Computer Fraud and Security 2020 Vol. 2020 Issue 6 Pages 6-8 | Does not mention cases of health corruption |
| 15 | Corruption and strengthening anti-corruption efforts in healthcare during the pandemic of Covid-19  . V. Teremetskyi, Y. Duliba, V. Kroitor, N. Korchak and O. Makarenko  . The Medico-legal journal 2021 Vol. 89 Issue 1 Pages 25-28 | Mentions cases of corruption from other countries |
| 16 | A land in agony: COVID-19, economic collapse, political corruption, and a deadly blast  A. N. Saliba and A. T. Taher  American Journal of Hematology 2021 Vol. 96 Issue 1 Pages E1-E2 | Mentions cases of corruption from other countries |
| 17 | International and national obligations to protect from the risks of pharmaceutical crime: The crime of counterfeit pharmaceutical products in the COVID-19 crisis  O. B. Layach  Systematic Reviews in Pharmacy 2020 Vol. 11 Issue 2 Pages 648-657 | Mentions cases of corruption from other countries |
| 18 | Dark Web Marketplaces and COVID-19: before the vaccine.   A. Bracci, M. Nadini, M. Aliapoulios, D. McCoy, I. Gray, A. Teytelboym, et al.  EPJ Data Science 2021 Vol. 10 Issue 1 | Mentions cases of corruption from other countries |
| 19 | Governmental actions to address COVID-19 misinformation  J. L. Pomeranz and A. R. Schwid  Journal of Public Health Policy 2021 | Mentions policies and laws to tackle corruption |
| 20 | Global Regulators Set Standards for Safe Vaccines.   B. M. Kuehn  JAMA - Journal of the American Medical Association 2020 Vol. 324 Issue 23 Pages 2356 | Mentions policies and laws to tackle corruption |
| 22 | An adaptive governance and health system response for the COVID-19 emergency  . M. Khan, P. Roy, I. Matin, M. Rabbani and R. Chowdhury  World Development 2021 Vol. 137 | Mentions policies and laws to tackle corruption |

1. <https://adfontesmedia.com/politico-bias-and-reliability/> [↑](#footnote-ref-1)
